# P-1170. Impact of Increased Severity of Illness on Outcomes in Complicated Urinary Tract Infections (cUTI) from ZEUS, a Randomized Controlled Trial (RCT) Evaluating IV Fosfomycin (IV-FOS)

**DOI:** 10.1093/ofid/ofaf695.1363

**Published:** 2026-01-11

**Authors:** Andrew Shorr, Keith S Kaye, Marya D Zilberberg, Judith N Steenbergen, Surya Chitra, Lauren Bjork, Mauricio Rodriguez

**Affiliations:** Medstar Washington Hospital Center; Rutgers Robert Wood Johnson Medical School, New Brunswick, NJ; EviMed Research Group, LLC, Goshen, Massachusetts; Scientific and Medical Affairs Consulting, LLC, Washington Crossing, Pennsylvania; Savio Group Analytics, Hockessin, Delaware; Meitheal Pharmaceuticals, Chicago, IL; Meitheal Pharmaceuticals, Chicago, IL

## Abstract

**Background:**

cUTIs remain a significant cause of hospital admissions and are an economic burden. IV-FOS is a first-in-class injectable epoxide antibiotic under development. A randomized trial (RCT) comparing IV-FOS to piperacillin-tazobactam (PIP-TAZ) in patients suffering from cUTIs demonstrated noninferiority with 64.7% of IV-FOS vs 54.5% of PIP-TAZ groups achieving a favorable response. We sought to compare outcomes within two subgroups: 1) patients with a high burden of chronic illness, and 2) patients with severe acute disease.

**Table 1.**
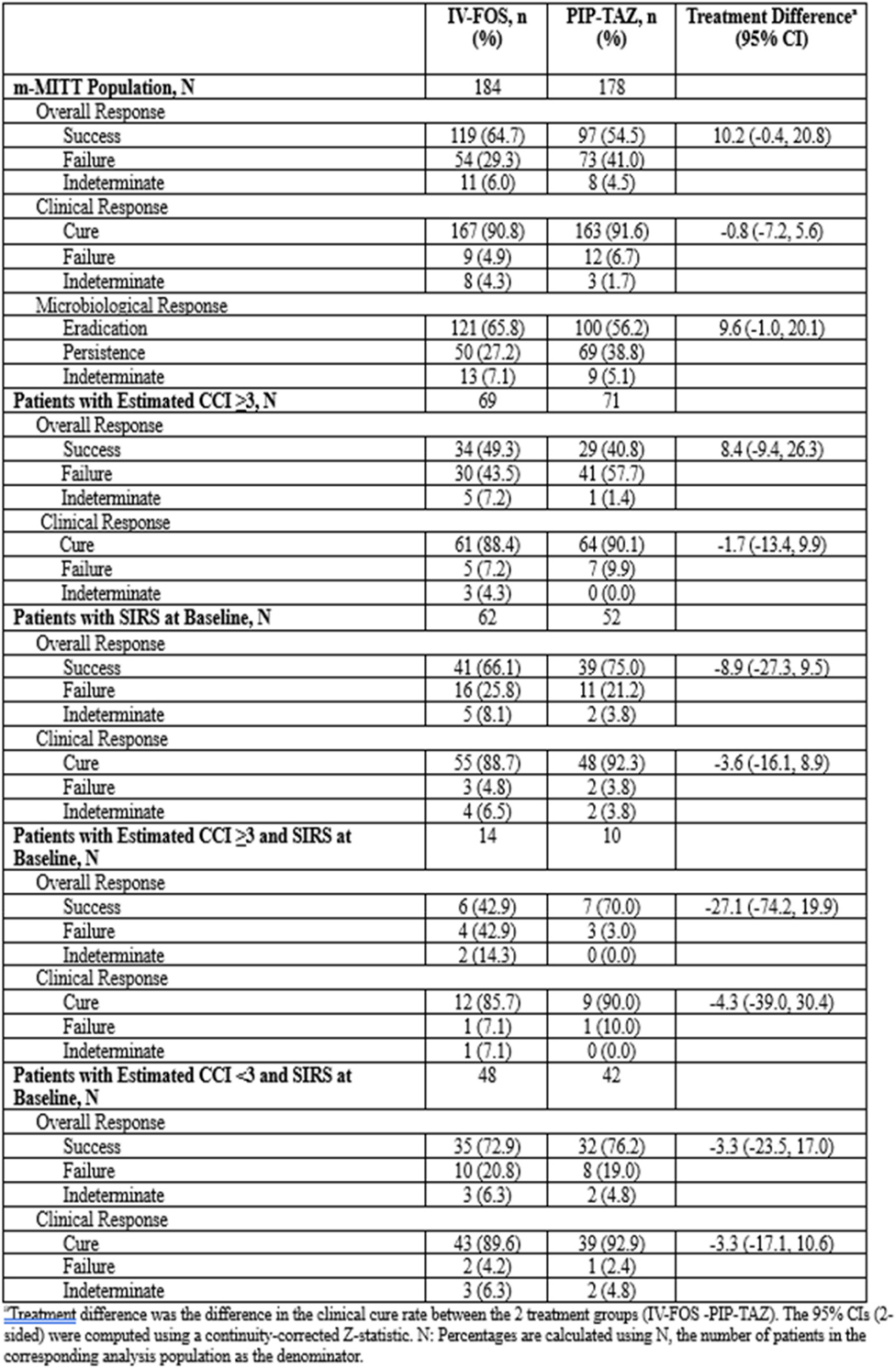
Clinical, Microbiological and Overall Response for Patients meeting SIRs Criteria or a CCI of ≥ 3 (m-MITT population)

**Methods:**

Two subgroup analyses of the phase 2/3 RCT of IV-FOS vs PIP-TAZ were conducted. A composite of clinical response, defined as both clinical success and microbiologic eradication, within the microbiologic-modified intention to treat (m-MITT) population, served as the primary endpoint. High chronic illness burden was defined as a Charlson co-morbidity index (CCI) > 3, while severe acute disease was based on meeting criteria for the systemic inflammatory response syndrome (SIRS).

**Results:**

Of 465 patients randomized, 362 (77.8%; 184 IV-FOS and 178 PIP-TAZ) constituted the m-MITT population. Within this group, 140 (38.7%; 37.5% IV-FOS vs 39.9% PIP-TAZ) had a CCI ≥ 3 and 114 (31.5%; 33.7% IV-FOS vs 29.2% PIP-TAZ) met the SIRS criteria. In unadjusted analyses, clinical response rates were similar between IV-FOS and PIP-TAZ in the two treatment groups among both patients with CCI ≥ 3 (49.3% IV-FOS vs 40.8% PIP-TAZ, p=NS) and those with SIRS (66.1% IV-FOS vs 75% PIP-TAZ, p=NS). For both subgroups, the individual components of the composites of clinical response as a function of study drug behaved similarly (Table 1).

**Conclusion:**

In this Phase 2/3 RCT in cUTI, subgroup analyses in patients with SIRS and those with high burdens of chronic illness demonstrated comparable composite outcomes between IV-FOS and PIP-TAZ treatments.

**Disclosures:**

Andrew Shorr, MD, MPH, MBA, Bioversys: Advisor/Consultant Keith S. Kaye, MD, MPH, AbbVie: Advisor/Consultant|GSK: Advisor/Consultant|Merck: Advisor/Consultant|Shionogi: Advisor/Consultant Marya D. Zilberberg, MD, MPH, Basilea Pharmaceutica: Grant/Research Support|Eagle Pharmaceuticals: Advisor/Consultant|Eagle Pharmaceuticals: Grant/Research Support|Shionogi: Advisor/Consultant Judith N. Steenbergen, PhD, AcurX: Advisor/Consultant|Basilea: Advisor/Consultant|Bioversys: Advisor/Consultant|Clarametyx: Advisor/Consultant|Eagle Pharmaceuticals: Advisor/Consultant|F2G: Advisor/Consultant|Genentech: Advisor/Consultant|Innoviva: Advisor/Consultant|Meitheal: Advisor/Consultant|Melinta: Advisor/Consultant|Neuraptive: Advisor/Consultant|Neuraptive: Advisor/Consultant|Roche: Advisor/Consultant|Wockhardt: Advisor/Consultant Lauren Bjork, PharmD, Meitheal: employee Mauricio Rodriguez, PharmD, MS-HEOR, BCCCP, BCIDP, Meitheal Pharmaceuticals: employee

